# Substrate Orientation-Dependent Synaptic Plasticity and Visual Memory in Sol–Gel-Derived ZnO Optoelectronic Devices

**DOI:** 10.3390/ma18184377

**Published:** 2025-09-19

**Authors:** Dabin Jeon, Seung Hun Lee, JungBeen Cho, Kyoung-Bo Kim, Sung-Nam Lee

**Affiliations:** 1 Department of IT & Semiconductor Convergence Engineering, Tech University of Korea, Siheung 15073, Republic of Korea; 2 Department of Semiconductor Engineering, Tech University of Korea, Siheung 15073, Republic of Korea; 3 Department of Materials Science & Engineering, Inha Technical College, Incheon 22212, Republic of Korea

**Keywords:** ZnO, sol–gel method, optoelectronic synapse, sapphire substrate, synaptic plasticity, visual memory, neuromorphic computing

## Abstract

We report Al/ZnO/Al optoelectronic synaptic devices fabricated on c-plane and m-plane sapphire substrates using a sol–gel process. The devices exhibit essential synaptic behaviors such as excitatory postsynaptic current modulation, paired-pulse facilitation, and long-term learning–forgetting dynamics described by Wickelgren’s power law. Comparative analysis reveals that substrate orientation strongly influences memory performance: devices on m-plane consistently show higher EPSCs, slower decay rates, and superior retention compared to c-plane counterparts. These characteristics are attributed to crystallographic effects that enhance carrier trapping and persistent photoconductivity. To demonstrate their practical applicability, 3 × 3-pixel arrays of adjacent devices were constructed, where a “T”-shaped optical pattern was successfully encoded, learned, and retained across repeated stimulation cycles. These results highlight the critical role of substrate orientation in tailoring synaptic plasticity and memory retention, offering promising prospects for ZnO-based optoelectronic synaptic arrays in in-sensor neuromorphic computing and artificial visual memory systems.

## 1. Introduction

Neuromorphic semiconductor devices that emulate the behavior of biological synapses have emerged as promising components for next-generation artificial intelligence (AI) hardware, particularly in applications such as in-sensor computing, intelligent vision, and edge-based learning systems [1,2,3]. Among various device platforms, optoelectronic synaptic devices have attracted considerable attention due to their ability to simultaneously sense and process optical signals, enabling direct interfacing with light-based neural inputs [4,5,6]. Zinc oxide (ZnO), a wide-bandgap (~3.3 eV) n-type semiconductor, is widely regarded as a suitable material for optoelectronic synaptic devices owing to its high transparency in the visible range, strong ultraviolet (UV) photoresponse, intrinsic oxygen vacancy-mediated defect states, and chemical stability [6,7,8,9]. Furthermore, the compatibility of ZnO with low-cost solution processes makes it advantageous for large-area, flexible, and scalable neuromorphic device fabrication [10,11,12]. Among various film deposition techniques, the sol–gel spin-coating method offers a cost-effective and straightforward approach for synthesizing ZnO thin films with controllable thickness and composition [13,14,15]. Compared to vacuum-based methods such as sputtering or pulsed laser deposition, sol–gel processing allows for precise doping control, uniform coating over large areas, and facile integration with various substrates without the need for complex equipment.

However, as-deposited sol–gel ZnO films typically exhibit poor crystallinity, necessitating high-temperature post-annealing to promote grain growth and enhance film quality [16,17]. During post-deposition thermal annealing, the crystallographic growth orientation of ZnO can be strongly influenced by the underlying substrate [7,18]. In particular, single-crystal sapphire substrates offer excellent thermal and chemical stability and exhibit distinct crystallographic surfaces, such as c-plane (0001) and m-plane (10-10), that interact differently with the growing ZnO lattice [19,20]. These anisotropic interfacial interactions guide the nucleation and alignment of ZnO grains during annealing, thereby influencing the dominant crystal orientation of the resulting film [21,22,23]. The crystallographic orientation of ZnO thin films plays a critical role in determining their optoelectronic synaptic performance. For example, c-axis-oriented ZnO films often exhibit strong band-edge emission and higher carrier mobility along the growth axis, while non-c-axis orientations such as (100) or (101) can alter defect densities and carrier dynamics [24,25]. These differences significantly affect short- and long-term synaptic plasticity, including paired-pulse facilitation (PPF) and memory retention, where long-term plasticity is influenced by persistent photoconductivity (PPC) [6,26,27,28]. In this study, we systematically investigate the effect of substrate orientation on the structural and neuromorphic properties of sol–gel-derived ZnO thin films. By comparing ZnO optoelectronic synaptic devices fabricated on c-plane and m-plane sapphire substrates, we demonstrate that crystallographic orientation modulates the synaptic plasticity and visual memory behavior of the devices. While most prior studies have focused on defect engineering (e.g., Al doping to optimize PPC and memory retention [13], ZnO-CNT nano composite formation to improve EPSC and PPF [29], Mg-doped memristor [30], and ZnO-NiO heterostructures [31]), the role of substrate orientation has remained relatively unexplored. Our results highlight that orientation-induced modulation of polarization fields, carrier dynamics, and PPC critically determines short-term plasticity, such as PPF, as well as long term memory behavior, including visual memory retention. This study emphasizes the role of interface engineering in neuromorphic materials and demonstrates that substrate selection is a critical factor in optimizing device performance for advanced optoelectronic computing systems.

## 2. Materials and Methods

ZnO thin films with a thickness approximately 100 nm were synthesized via a sol–gel spin-coating process on single-crystal sapphire substrates with two different orientations: c-plane (0001) and m-plane (10-10). The precursor solution was prepared by dissolving zinc acetate dihydrate and monoethanolamine in 2-methoxyethanol at a 1:1 molar ratio. The solution was stirred at 1000 rpm and 100 °C for 30 min to ensure complete mixing and chelation. The resulting sol was spin-coated onto the sapphire substrates using 5 drops per cycle at 6000 rpm for 30 s. Each deposited layer was pre-baked at 200 °C for 5 min to remove residual solvents. After completing the desired number of coating cycles, the films were subjected to rapid thermal annealing at 900 °C for 1 min in ambient air to improve crystallinity. This process resulted in polycrystalline ZnO thin films with orientation-dependent grain structure and optical properties. To evaluate the optoelectronic synaptic behavior, metal–semiconductor–metal (MSM) structured devices were fabricated using the ZnO films as the active channel layer. Aluminum (Al) electrodes with a thickness of 50 nm were deposited by thermal evaporation through a shadow mask to define a rectangular sensing area of 50 × 825 μm^2^. The symmetric electrode configuration was aligned along the long axis of the ZnO film to ensure uniform current flow under UV excitation.

The surface morphology and grain size of the ZnO thin films were characterized using atomic force microscopy (AFM). Crystallographic properties and texture orientations were analyzed using X-ray diffraction (XRD) with θ/2θ scans. UV–visible absorption spectroscopy was used to evaluate the optical transmittance and estimate the optical bandgap via Tauc plots. Photoluminescence (PL) spectroscopy was performed at room temperature to investigate near-band-edge and deep-level emissions. Raman spectroscopy was used to probe phonon modes and assess crystallinity and stress effects in the films. The electrical and synaptic performance of the ZnO MSM devices was characterized using an HP4155A semiconductor parameter analyzer (Keysight, Santa Rosa, CA, USA). The excitatory postsynaptic current (EPSC) was measured under a constant bias voltage of 1.0 V. Synaptic potentiation and depression were emulated by applying pulsed UV light (265 nm, 66 µW cm^−2^) to the devices and monitoring the transient current response. Short-term plasticity was evaluated via PPF, applying two consecutive light pulses with varying inter-spike intervals and calculating the facilitation index (A_2_/A_1_ × 100%). Long-term synaptic behavior was assessed by applying repeated light pulses and analyzing the learning (EPSC buildup) and forgetting (EPSC decay) characteristics. For visual memory simulations, a 3 × 3 array of MSM synaptic devices was selectively illuminated with patterned light pulses to encode image data. The EPSC levels of each pixel were recorded before and after light exposure to evaluate image retention and memory reinforcement under repeated learning cycles.

## 3. Results and Discussion

### 3.1. Optical and Structural Properties of ZnO Thin Films Grown by C and M-Plane Sapphire Using Sol–Gel Method

**Figure 1:**
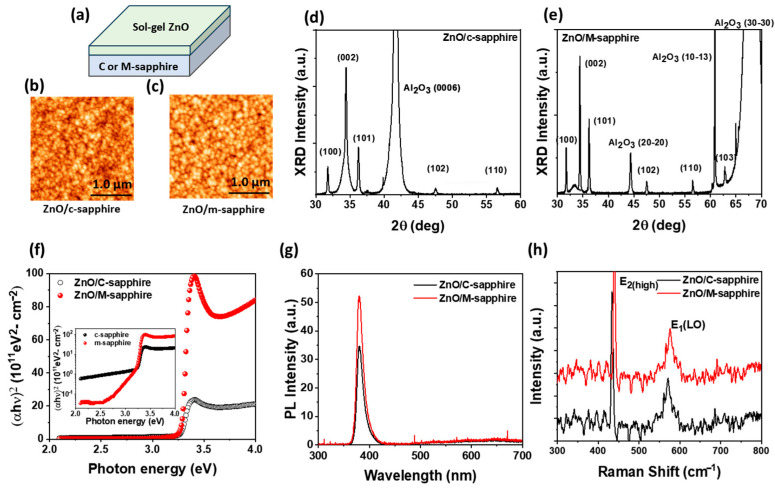
(**a**) Schematic illustration of sol–gel-derived ZnO thin films deposited on c-plane and m-plane sapphire substrates. AFM surface images of ZnO films on (**b**) c-plane and (**c**) m-plane sapphire. XRD θ/2θ scans of ZnO films on (**d**) c-plane and (**e**) m-plane sapphire. (**f**) UV–Vis absorption spectra as a function of photon energy for ZnO films on both substrates; the inset shows the spectra on a logarithmic scale to emphasize deep-level absorption. (**g**) Photoluminescence (PL) and (**h**) Raman spectra of ZnO thin films on c-plane and m-plane sapphire substrates.

Figure 1a presents a schematic illustration of sol–gel-derived ZnO thin films on c-plane and m-plane sapphire substrates, along with corresponding AFM surface morphology images in Figure 1b,c As illustrated in Figure 1b,c, the AFM surface images reveal a distinct difference in surface grain morphology between the two samples. The ZnO film grown on the c-plane sapphire exhibits a smoother surface with relatively small grains (average grain size ≈ 83 nm) and a lower surface roughness (RMS = 8.24 nm), which can be attributed to the alignment of the polar (002) ZnO plane with the c-plane sapphire, promoting vertical columnar growth and suppressing lateral grain expansion, thereby yielding a more compact and uniform surface. In contrast, the film grown on the m-plane sapphire displays larger grains (average grain size ≈ 99 nm) with more pronounced grain boundaries and a higher surface roughness (RMS = 11.6 nm), due to the increased freedom of growth directions on nonpolar or semipolar orientations, which facilitates lateral crystallite growth and results in a rougher surface morphology [7]. To analyze the crystallographic orientation of the films, XRD θ/2θ scans were performed, as shown in Figure 1d (c-plane) and Figure 1e (m-plane). Both samples exhibit multiple ZnO diffraction peaks corresponding to the (100), (002), (101), (102), and (110) planes, confirming the formation of polycrystalline ZnO films with various crystallographic orientations. In the ZnO/c-sapphire sample, the (002) ZnO peak is not only the most intense but also broader than other reflections, indicating a dominant c-axis growth accompanied by a certain degree of mosaic spread. Quantitative peak area analysis revealed that the polar ZnO (002) plane accounts for 71.3% of the total ZnO diffraction intensity in the ZnO/c-sapphire film, compared to 43.8% for the ZnO/m-sapphire film, indicating a much stronger c-axis preferential orientation on the c-plane substrate. This is further supported by the Harris Texture Coefficient (TC) of the (002) plane [32], which is 1.907 for ZnO/c-sapphire versus 1.505 for ZnO/m-sapphire, while the nonpolar and semipolar planes collectively show higher TC values in the m-plane sample. Figure 1f presents the (αhν)^2^–Photon energy plots of ZnO thin films grown on c-plane and m-plane sapphire substrates. The optical bandgap values, extracted from the Tauc plot, are approximately 3.3 eV for both films, which is consistent with the intrinsic ZnO bandgap [6,7]. The m-plane ZnO shows a steeper absorption edge and stronger absorption above the bandgap, attributed to suppressed internal polarization fields and reduced defect scattering, thereby enhancing photon absorption efficiency. In contrast, the c-plane ZnO exhibits relatively higher sub-bandgap absorption, which suggests a greater density of defect-related deep-level states. This is further illustrated in the inset of Figure 1f, where the absorption spectra are plotted on a logarithmic scale to highlight deep-level absorption features. Figure 1g shows the room-temperature PL spectra, revealing radiative recombination characteristics of ZnO films grown on c-plane and m-plane sapphire substrates. The ZnO film on the m-plane substrate exhibits a significantly stronger near-band-edge (NBE) emission, while the deep-level emissions are nearly comparable for both samples. This suggests superior crystallinity and reduced grain boundary defects in the m-plane sample, likely due to its larger grain size and more coherent crystalline domains. In addition, the m-plane orientation suppresses polarization-field-induced effects that are prominent in c-plane ZnO, leading to reduced band bending and minimized emission intensity reduction or spectral shift [33,34]. This suppression minimizes band bending within the active region, enhancing the overlap of electron and hole wavefunctions. The increased wavefunction overlap facilitates radiative recombination, leading to stronger NBE emissions. Consequently, the improved optical transition probability and reduced non-radiative loss pathways contribute to the enhanced PL intensity in the m-plane ZnO film.

Figure 1h presents the micro-Raman spectra of the ZnO films deposited on c-plane and m-plane sapphire substrates. Compared with the ZnO film on c-plane sapphire, the ZnO film on m-plane sapphire exhibits a clear blue shift in both the E_2_ (high) and E_1_ modes. This shift can be attributed to compressive strain arising from the anisotropic lattice mismatch between ZnO and the m-plane sapphire substrate [35]. The induced compressive stress shortens the Zn–O bond length, thereby increasing the phonon vibration frequency and leading to the observed blue shift. In conjunction with AFM analysis, which reveals that the m-plane ZnO film forms larger grains with improved crystallinity, these results suggest that the m-plane sapphire not only promotes enhanced structural quality but also alters the residual strain state, ultimately modifying the phonon dynamics of the ZnO lattice.

### 3.2. Electrical and Optoelectronic Properties of ZnO Thin Films on Different Sapphire Substrates

**Figure 2:**
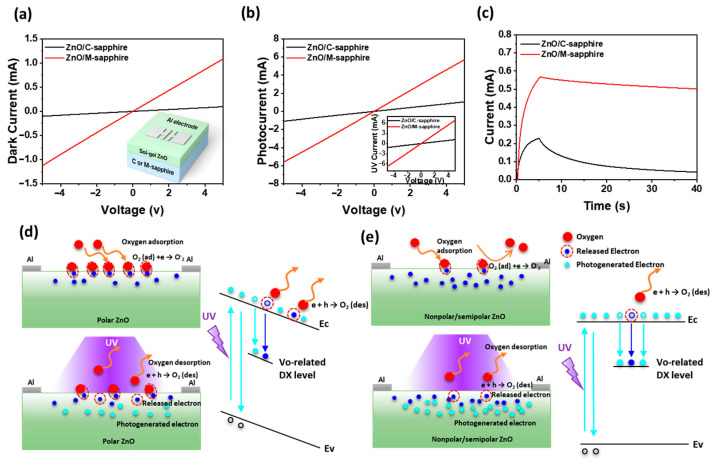
(**a**) Dark and (**b**) photocurrent–voltage characteristics of Al/ZnO/Al optoelectronic synaptic devices fabricated on c-plane and m-plane sapphire substrates, with insets showing the device schematic (**a**) and corresponding UV current–voltage curves. (**c**) Photocurrent as a function of applied bias. PPC decay following 5 s UV illumination. Schematic illustrations and band diagrams of oxygen adsorption/desorption and carrier generation in (**d**) polar-dominant ZnO film on c-sapphire and (**e**) nonpolar/semipolar-dominant ZnO film on m-sapphire.

The schematic structure of the Al/ZnO/Al optoelectronic synaptic device, comprising Al top electrodes deposited onto ZnO films grown on either c-plane or m-plane sapphire substrate are shown in the inset of Figure 2a. This horizontal Al/ZnO/Al structure enables a reliable analysis of the photoresponse characteristics under UV illumination. Figure 2a shows the current–voltage (I–V) characteristics of the Al/ZnO/Al devices under dark and UV illumination. At an operating voltage of 1.0 V, the dark current of the ZnO film grown on c-plane sapphire (19 µA) is significantly lower than that of the film on m-plane sapphire (217 µA), indicating that the dominant polar ZnO (002) orientation on c-plane sapphire possesses a lower intrinsic carrier concentration than the dominant nonpolar/semipolar orientations on m-plane sapphire, likely due to differences in crystal defect density. Upon UV illumination (inset of Figure 2b), the photocurrent of the device on m-plane sapphire exceeds that of the c-plane sample, which can be attributed to the suppression of internal polarization field in nonpolar/semipolar ZnO, resulting in enhanced optical transitions, as also evidenced in Figure 1g. Figure 2b shows the photocurrent (I_ph_ = I_UV_ − I_dark_) of Al/ZnO/Al devices fabricated on c-plane and m-plane sapphire substrates as a function of applied voltage. The Al/ZnO/Al device fabricated on m-plane sapphire exhibits a substantially higher photocurrent compared to its c-plane counterpart, which is ascribed to more efficient photocarrier generation and improved charge transport characteristics facilitated by the nonpolar/semipolar orientation. This superior performance is attributed to the larger grain size, higher surface roughness, and improved optical properties of the ZnO film on m-plane sapphire (Figure 1b,c,g), which facilitate more efficient light absorption and carrier mobility. Figure 2c presents the temporal photoresponse (I–t) curves measured under periodic UV illumination. The Al/ZnO/Al device on the m-plane sapphire shows a more pronounced and stable increase in current upon light exposure, along with a slower decay in the dark. This suggests stronger PPC behavior, which is likely due to the reduced recombination rate of photogenerated carriers in the presence of fewer grain boundaries and trap sites [36]. In nonpolar/semipolar ZnO grown on m-sapphire, the absence of a strong internal polarization field facilitates more efficient separation of photogenerated carriers and prolong carrier lifetime. As illustrated Figure 2d, although polar-dominant ZnO film on c-sapphire exhibit stronger oxygen adsorption in the dark due to their polarity and surface charge imbalance, the presence of a strong internal electric field accelerates electron–hole recombination, thereby limiting the photocurrent response. In contrast, as shown in Figure 2e, nonpolar/semipolar-dominant ZnO film grown on m-sapphire exhibit relatively weaker oxygen adsorption compared to polar (002) surfaces due to the reduced surface polarity. However, the suppression of the internal polarization field in these orientations leads to flatter band alignment and longer carrier lifetimes, reducing recombination losses and enhancing PPC. As supported by prior reports [37], PPC in oxides originates from oxygen vacancy state (V_o_) that are photo-ionized to shallow donor levels; the resulting outward bond relaxation forms an energy barrier for recombination, thereby sustaining photoconductivity. In out devices, the weaker polarization field in nonpolar/semipolar ZnO further slows recombination of these ionized vacancy states, enabling more persistent carrier conduction. Thus, even though fewer oxygen molecules are initially adsorbed, the overall PPC is significantly enhanced [7,38]. Upon UV illumination, in nonpolar/semipolar ZnO, photogenerated holes efficiently react with the comparatively smaller number of adsorbed oxygen ions (O_2_), yet the extended carrier lifetime ensures more effective oxygen desorption and a greater release of electrons into the conduction band. The reduced internal field prolongs carrier lifetime, enabling a more sustained release of free carriers and a stronger PPC response. Therefore, the combination of moderated oxygen adsorption, vacancy-related PPC dynamics, and polarization-field suppression explains the markedly enhanced PPC in nonpolar/semipolar-dominant ZnO films compared to polar-dominant ZnO film, where stronger oxygen adsorption but faster recombination suppresses the PPC response.

### 3.3. Unified Synaptic Plasticity Behavior in Sol–Gel-Derived ZnO Devices: PPF and STDP-like Timing-Dependent Synaptic Modulation

**Figure 3:**
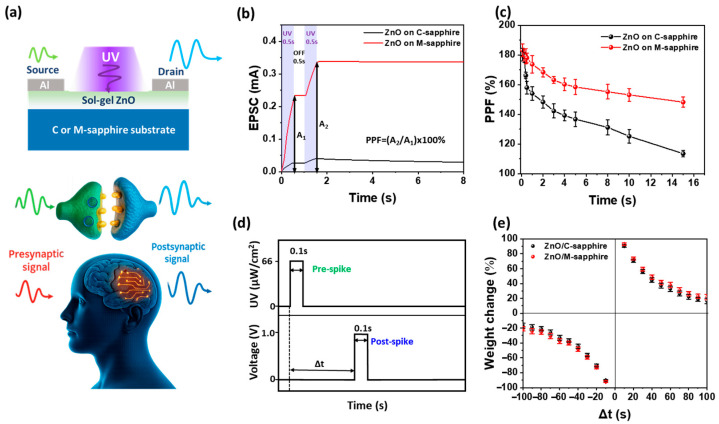
(**a**) Schematic illustration of UV-stimulated ZnO synaptic device and visual memory analogy. (**b**) Paired-pulse EPSC responses and (**c**) PPF versus Δt for Al/ZnO/Al optoelectronic synaptic devices on c-plane and m-plane devices, highlighting longer τ_1_ and τ_2_ for m-plane. (**d**) UV exposure and voltage profile used for STDP-like timing-dependent measurement and (**e**) synaptic weight change (ΔW/W_0_) versus Δt, showing STDP-like bidirectional modulation in ZnO optoelectronic synaptic devices formed on c-plane and m-plane sapphire substrates.

To investigate the short-term synaptic plasticity (STP) of the sol–gel-derived Al/ZnO/Al optoelectronic synaptic devices, we performed PPF and STDP-like time-dependent synaptic weight modulation measurements under sequential UV light stimulations. As schematically illustrated in Figure 3a, the device operation under UV excitation emulates the short-term memory behavior of biological synapses, analogous to the visual pathway in which optical stimuli are processed by the human eye and transmitted to the brain for image storage. The observed responses reproduce the dynamic nature of synaptic transmission, where the second EPSC is influenced by the timing and strength of the preceding stimulus. Figure 3b presents the EPSC responses of sol–gel-derived Al/ZnO/Al optoelectronic synaptic devices fabricated on c-plane and m-plane sapphire substrates under paired 0.5 s UV (265 nm) pulses separated by a 0.5 s off interval. The Al/ZnO/Al device on m-plane sapphire exhibited an EPSC amplitude approximately seven times higher than that of its c-plane counterpart and a slower decay in the dark. These enhancements are attributed to the nonpolar/semipolar orientation of ZnO on m-plane sapphire, which provides abundant surface oxygen adsorption/desorption sites, suppresses the polarization effect, and incorporates defect-related DX states that prolong photocarrier lifetime, thereby amplifying and sustaining the synaptic response [6]. The PPF index, defined as [6,39]

PPF = (A_2_/A_1_) × 100% (1)

was evaluated as a function of the inter-spike interval (Δt) for ZnO synaptic devices fabricated on c-plane and m-plane sapphire substrates (Figure 3c). At a short interval of Δt = 0.1 s, the c-plane and m-plane devices exhibited PPF values of 180.2% and 183.2%, respectively, indicating slightly higher facilitation in the m-plane sample. As Δt increased to 15 s, the PPF decreased to 113.5% for the c-plane device, while the m-plane device retained a significantly higher value of 148.2%, confirming superior photocarrier retention in the latter. The decay of PPF with increasing Δt was fitted using a double-exponential function [13],

PPF(Δt) = C_1_ exp(−Δt/τ_1_) + C_2_ exp(−Δt/τ_2_) (2)

where τ_1_ and τ_2_ correspond to the fast and slow decay time constants, respectively. For the c-plane ZnO device, τ_1_ ≈ 0.3 s and τ_2_ ≈ 13.6 s, whereas for the m-plane ZnO device, τ_1_ ≈ 1.6 s and τ_2_ ≈ 16.7 s, indicating that the m-plane sample possesses longer decay times for both fast and slow processes. This suggests superior charge retention and slower recombination in the m-plane device. The much shorter τ_1_ compared to τ_2_ reflects the rapid relaxation of free photocarriers, while the long τ_2_ is attributed to the persistent release of carriers trapped at deep-level defect states and surface adsorbates [40]. This improvement is attributed to the nonpolar/semipolar nature of the m-plane sapphire substrate, which influences surface oxygen adsorption/desorption, reduces polarization-related internal fields, and mitigates defect-related non-radiative recombination pathways. Figure 3d,e present the UV and voltage input profile and the corresponding STDP-like time-dependent synaptic weight modulation of the Al/ZnO/Al synaptic devices fabricated on c-plane and m-plane sapphire substrates, respectively. Although the weight change curves exhibit a bidirectional modulation with positive values for Δt > 0 and negative values for Δt < 0, these results should be regarded as short-term STDP-like timing-dependent responses rather than conclusive evidence of long-term STDP [41]. The observed modulation reflects the immediate influence of relative spike timing on synaptic weight, but additional experimental validation involving persistent potentiation and depression would be necessary to confirm genuine long-term STDP behavior. A 0.1 s UV pre-spike was applied, followed by an inter-spike interval Δt and a 0.1 s electrical post-spike at 1.0 V; the synaptic weight change was evaluated as ΔW/W_0_ (%). Both ZnO devices on c-plane and m-plane sapphires exhibit a symmetric, bidirectional STDP-like timing-dependent window, with potentiation (positive weight change) for Δt > 0 and depression (negative weight change) for Δt < 0. These timing-dependent responses demonstrate bidirectional weight modulation; however, they should be regarded as short-term STDP-like characteristics rather than conclusive evidence of long-term plasticity. The Al/ZnO/Al optoelectronic synaptic device on m-plane sapphire substrate yields a larger weight change at small ∣Δt∣, indicating stronger spike-timing sensitivity and more efficient carrier modulation. This enhancement is attributed to the nonpolar/semipolar-dominant orientation of ZnO on m-sapphire, where reduced polarization effects and surface oxygen adsorption/desorption dynamics promote greater photocarrier participation during paired spikes.

**Figure 4:**
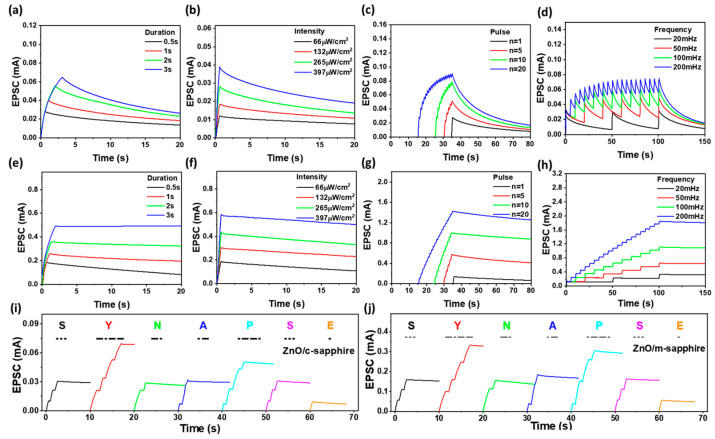
Long-term synaptic plasticity of sol–gel-derived ZnO optoelectronic synaptic devices fabricated on c-plane (**a**–**d**) and m-plane (**e**–**h**) sapphire substrates under varying UV stimulation parameters: (**a**,**e**) pulse duration (0.5–3.0 s), (**b**,**f**) light intensity (66–397 μW cm^−2^), (**c**,**g**) pulse count (1–20), and (**d**,**h**) pulse frequency (20–200 mHz). Optical encoding and retention of the Morse-coded word “SYNAPSE” for Al/ZnO/Al optoelectronic synaptic devices on (**i**) c-plane and (**j**) m-plane sapphire substrates.

Figure 4 presents the long-term synaptic plasticity characteristics of sol–gel-derived ZnO optoelectronic synaptic devices fabricated on c-plane (Figure 4a–d) and m-plane (Figure 4e–h) sapphire substrates under various UV stimulation protocols: (Figure 4a,e) UV light pulse duration ranging from 0.5 to 3.0 s, (Figure 4b,f) light intensity from 66 to 397 μW cm^−2^, (Figure 4c,g) number of pulses from 1 to 20, and (Figure 4d,h) pulse frequency from 20 to 200 mHz. In both substrate types, stronger or longer UV stimuli produced higher EPSCs and longer retention times. However, the Al/ZnO/Al optoelectronic synaptic device on m-plane sapphire consistently exhibited higher EPSCs and significantly slower decay rates under the same conditions, indicating more pronounced PPC and superior long-term potentiation characteristics compared to Al/ZnO/Al device on c-plane sapphire. This enhancement is attributed to the reduced internal polarization fields, improved crystallinity, and larger grain sizes of m-plane ZnO films, which promote more efficient photocarrier generation, trapping, and storage [7,38]. After confirming the enhancement of EPSC and retention behavior under four types of stimulation, the devices were further evaluated for information storage using Morse code patterns. Figure 4i,j present this functional image-retention analysis, where the word “SYNAPSE” was encoded through dot and dash UV pulses and projected onto the devices. Both Al/ZnO/Al optoelectronic devices on c-plane and m-plane sapphires successfully stored and reproduced the optical pattern, confirming their capacity for complex spatiotemporal information encoding. Consistent with the EPSC and retention results, the Al/ZnO/Al optoelectronic device on m-plane sapphire demonstrated markedly higher EPSC amplitude and longer decay times for the same stimulus, reflecting stronger carrier trapping and suppressed non-radiative recombination. These properties directly translate into more robust and persistent image retention, which is critical for neuromorphic vision and in-sensor computing applications. Taken together, the combination of pulse-dependent EPSC enhancement (Figure 4a–h) and reliable image retention (Figure 4i,j) highlights the superior synaptic performance of nonpolar/semipolar-dominant ZnO optoelectronic synaptic device on m-plane sapphire. This synergy between material structure and optical memory function suggests that substrate engineering is an effective route for optimizing neuromorphic optoelectronic systems.

### 3.4. Learning–Forgetting Dynamics and Visual Memory in ZnO-Based Optoelectronic Synaptic Devices

**Figure 5:**
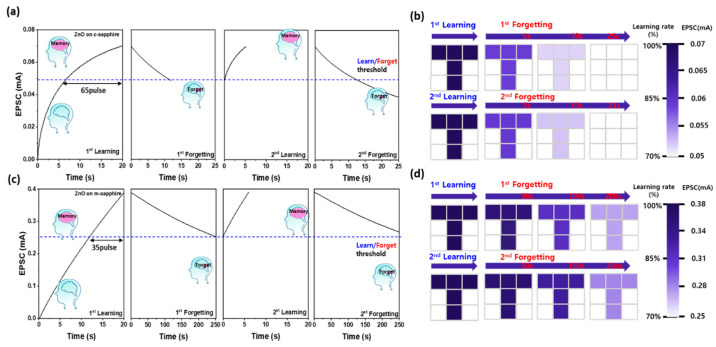
Learning–forgetting experience process under UV pulse stimulation for the Al/ZnO/Al optoelectronic synaptic devices on (**a**) c-plane and (**c**) m-plane sapphire substrates. Visual memory retention mapping of 3 × 3-pixel arrays composed of nine adjacent Al/ZnO/Al optoelectronic synaptic devices fabricated on (**b**) c-plane and (**d**) m-plane sapphire substrates, recorded during the first and second learning–forgetting cycles.

Figure 5a,c illustrates the comparative learning–forgetting dynamics of Al/ZnO/Al optoelectronic synaptic devices fabricated on c-plane and m-plane sapphire substrates under repetitive UV stimulation, respectively. Both devices were subjected to 265 nm UV pulses (pulse width 0.1 s, duty cycle 50%) for 100 cycles to induce 100% learning, followed by a natural decay phase without illumination. This learn–forget sequence was repeated twice to evaluate retention behavior upon re-learning. The learn/forget threshold, defined as 70% of the maximum excitatory postsynaptic current (EPSC), is indicated by the blue dotted lines in Figure 5a,c. For the Al/ZnO/Al optoelectronic device on c-plane sapphire, EPSC increased rapidly during the initial UV pulses but exhibited a noticeable saturation trend, requiring ~65 pulses to reach 100% EPSC from the threshold. In contrast, the Al/ZnO/Al optoelectronic device on m-plane sapphire demonstrated a nearly linear EPSC growth up to the 100th pulse, requiring only ~35 pulses to reach full EPSC, indicating a more efficient photo-induced carrier accumulation. This enhanced learning efficiency is attributed to reduced internal polarization fields and improved photocarrier transport along the m-plane orientation, which mitigates saturation effects observed in the c-plane configuration. Retention analysis revealed significant differences between the two substrates. The Al/ZnO/Al optoelectronic device on c-plane sapphire showed relatively short forgetting times, with the decay from maximum EPSC to the threshold occurring in 11.7 s during the first cycle and 12.4 s during the second cycle. Conversely, the Al/ZnO/Al optoelectronic device on m-plane sapphire exhibited remarkably slower decay, with forgetting times of 250 s and 290 s for the first and second cycles, respectively. This indicates that both devices benefit from repeated learning through extended retention, but the Al/ZnO/Al optoelectronic device on m-plane sapphire offers substantially longer memory persistence, consistent with its stronger PPC. These observations were quantitatively supported by fitting the decay curves to Wickelgren’s power law [29,41],

I = λ(1 + βt)^−ψ^ (3)

where I is the memory intensity, t is the decay time, λ is the initial memory strength (i.e., the long-term memory value at t = 0 s), β represents the rate constant of memory decay (larger β indicates a faster initial rate of forgetting), and ψ denotes the forgetting exponent, where a smaller ψ corresponds to a slower forgetting rate and thus enhanced long-term memory retention. For the Al/ZnO/Al optoelectronic synaptic device on c-plane sapphire, ψ was 0.896 in the first cycle and 0.750 in the second cycle, indicating improved long-term retention after repeated learning process. In contrast, the ψ value of the Al/ZnO/Al optoelectronic device on m-plane sapphire decreased from 0.701 in the first cycle to 0.525 in the second cycle, indicating that the forgetting rate becomes slower after re-learning process, allowing the learned information to be retained for a longer period. Moreover, compared to the Al/ZnO/Al optoelectronic synaptic device fabricated on c-plane sapphire, the consistently smaller ψ values for the Al/ZnO/Al optoelectronic synaptic device on m-plane sapphire demonstrate its slower forgetting rate and enhanced long-term memory retention. The visual memory capability of the devices was further evaluated using 3 × 3-pixel arrays fabricated by integrating nine adjacent Al/ZnO/Al optoelectronic synaptic devices (Figure 5b,d), where a “T”-shaped optical pattern was encoded with the same UV pulse parameters. All devices in the array exhibited consistent synaptic responses, indicating good reproducibility and low variability across the array, which supports the reliability of the sol–gel fabrication method. Both devices reproduced the intended “T” pattern more distinctly after the second learning cycle, demonstrating the reinforcement effect. 

However, while the Al/ZnO/Al optoelectronic device on c-plane sapphire lost pattern visibility within ~25 s, the Al/ZnO/Al optoelectronic device on m-plane sapphire retained a high-contrast image for over 200 s, confirming its superior long-term potentiation and retention. Overall, the results demonstrate that the m-plane sapphire substrate significantly enhances both learning efficiency and retention in sol–gel-derived ZnO synaptic devices. The improvement is ascribed to the reduced spontaneous polarization and more favorable carrier dynamics along the nonpolar/semipolar-dominant ZnO planes, which facilitate sustained photocarrier trapping and slower recombination, thereby prolonging the memory state. These findings provide important insight into substrate-orientation engineering for high-performance optoelectronic synaptic devices with enhanced long-term memory characteristics.

## 4. Conclusions

We developed Al/ZnO/Al optoelectronic synaptic devices on c-plane and m-plane sapphire substrates and systematically examined their neuromorphic functionality. The devices exhibited key synaptic behaviors, including excitatory postsynaptic current modulation, paired-pulse facilitation, and learning–forgetting processes described by the Wickelgren power law. Substrate orientation played a decisive role in tuning memory retention: c-plane devices showed accelerated decay after repeated learning, while m-plane devices demonstrated stronger reinforcement with slower forgetting, indicating superior long-term stability. To further validate their capability, 3 × 3-pixel arrays of adjacent devices were fabricated and tested with a “T”-shaped optical pattern. The arrays successfully reproduced learning–forgetting cycles and partially retained information after repeated training, confirming their potential for pattern recognition and visual memory. These findings highlight crystallographic orientation as a key parameter for tailoring synaptic plasticity, advancing the design of oxide-based optoelectronic synaptic arrays for in-sensor computing and artificial visual systems.

## Data Availability

The data presented in this study are available on request from the corresponding author. The data are not publicly available due to privacy concerns.
